# MiR‐340‐5p alleviates neuroinflammation and neuronal injury via suppressing STING in subarachnoid hemorrhage

**DOI:** 10.1002/brb3.2687

**Published:** 2022-08-11

**Authors:** Ning Song, Rong Song, Peiliang Ma

**Affiliations:** ^1^ Department of Emergency The 940th Hospital of Joint Logistics Support force of Chinese People's Liberation Army Lanzhou Gansu China; ^2^ Department of Oral Medicine Lanzhou University Dental Hospital Lanzhou Gansu China; ^3^ Department of Orthopedics Lanzhou PLA 96604 Military Hospital Lanzhou Gansu China

**Keywords:** early brain injury, miR‐340‐5p, neuroinflammation, STING, subarachnoid hemorrhage

## Abstract

**Background:**

Subarachnoid hemorrhage (SAH) is a severe acute neurological disorder. SAH causes neuroinflammation and leads to early brain injury (EBI) and secondary injury. MicroRNAs are crucial regulators in a variety of neurological diseases. This study was performed to decipher how miR‐340‐5p functions in SAH.

**Methods:**

An experimental mouse model with SAH was established by the intravascular perforation, and the in vitro SAH model was constructed by exposing cocultured primary neurons and microglia to oxyhemoglobin. After overexpression of miR‐340‐5p in mice, the neurobehavioral disorders were evaluated by Garcia test; brain edema was evaluated by wet–dry method; blood–brain barrier (BBB) damage was detected with Evan's blue staining; levels of inflammatory cytokines were detected with enzyme‐linked immunosorbent assay. After miR‐340‐5p was transfected in to microglia, Iba‐1 expression was detected by Western blot, and neuronal apoptosis were detected with flow cytometry. The targeting relationship between miR‐340‐5p and STING was verified by dual‐luciferase reporter gene assay and RNA immunoprecipitation assay.

**Results:**

MiR‐340‐5p was significantly inhibited in the brain tissues of mice with SAH and microglia of SAH model, and neurological impairment, brain edema, BBB injury, and neuroinflammation were significantly alleviated in mice after overexpressing miR‐340‐5p. STING was identified as a target of miR‐340‐5p, and STING overexpression could counteract the effects of miR‐340‐5p overexpression on neurons.

**Conclusion:**

MiR‐340‐5p can attenuate EBI caused by SAH‐induced neuroinflammation by inhibiting STING.

## 1 INTRODUCTION

Subarachnoid hemorrhage (SAH) is a severe neurological disease with high morbidity and mortality (Balbi et al., 2020; Kamp et al., 2020). Early brain injury (EBI) after SAH may be the main driver of poor prognosis (Dou et al., 2017; Helbok et al., 2015). Neuroinflammation is one of the significant pathological processes of EBI (Liu et al., 2019). After SAH, the microglia are activated and excessive inflammatory cytokines are produced and released, which leads to inflammatory response and aggravates neurological impairment (Li & Zhou, 2022; Suzuki, 2019; Zheng et al., 2020). Therefore, relieving neuroinflammation may improve the prognosis of the patients SAH.

MicroRNAs (miRNAs) are widespread RNA molecules of approximately 21–23*nt* in eukaryotes (Bartel, 2004), which inhibits the translation of mRNA via binding to the 3′‐untranslated region (UTR) of the target mRNA (Bartel, 2009). Reportedly, miRNAs are crucial regulators in many neurological diseases (Deverman et al., 2018). For example, miR‐7 and miR‐22 are potential to alleviate Parkinson's disease (Salama et al., 2020). MiR‐212‐5p can probably mitigate the pathological changes after traumatic brain injury (Xiao et al., 2019). Also, many miRNAs, such as miR‐124 and miR‐193b‐3p, are also found to be dysregulated during the pathogenesis of SAH (Chen et al., 2020; Lai et al., 2020). Reportedly, miR‐340‐5p can reduce cerebral hemorrhage and spinal cord injury‐induced nerve injury and play a protective role by inhibiting neuroinflammation (Qian et al., 2020; Zhou et al., 2020), but its expression and function in SAH have not been reported yet.

Stimulator of interferon genes (STING) is also known as transmembrane protein 173 (TMEM173) and MPYS/MITA/ERIS. STING promotes type‐I interferon (IFN)‐related inflammatory responses and regulates other biological processes such as autophagy, cell survival, and senescence (Wan et al., 2020). STING pathway is involved in regulating the pathogenesis of infection, inflammatory diseases, and tumorigenesis by interacting with other innate immune pathways (Kwon & Bakhoum, 2020; Ma et al., 2020; Wen & Li, 2020). Some studies have shown that inhibition of STING can potentially block the progression of Parkinson's disease and Huntington's disease by inhibiting neuroinflammation (Jauhari et al., 2020; Sliter et al., 2018).

In the present study, we constructed in vivo and in vitro SAH models, and we report that miR‐340‐5p expression is inhibited after SAH, which regulates the inflammatory response and microglia activation after SAH. It is further confirmed that miR‐340‐5p exerts its neuroprotective effects through the regulation of STING in EBI of SAH.

## 2 MATERIALS AND METHODS

### 2.1 SAH model in mice

**FIGURE 1:**
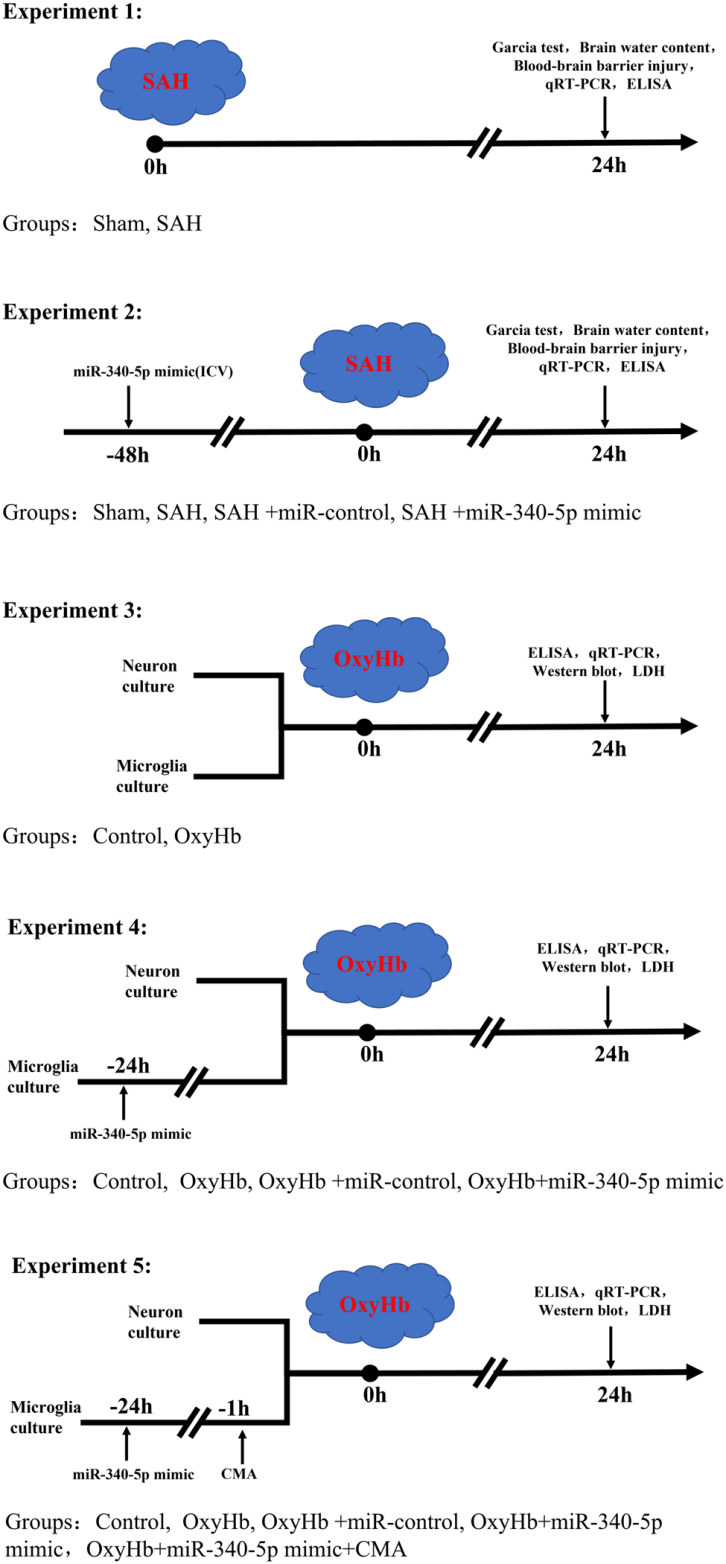
Experimental design of the present work

The experiments were performed with adult male C57BL/6 mice (25–30 g, Animal Center of Chinese Academy of Sciences, Shanghai, China). All procedures were endorsed by the Ethics Committee of Lanzhou PLA 96604 Military Hospital, and performed according to the National Institutes of Health and American Physiological Society Guidelines for the Care and Use of Laboratory Animals. The experimental design was shown in Figure 1. First, the mice were randomly grouped into two group: Sham group and SAH group (18 alive mice in each group). The establishment of mouse SAH model was carried out according to a previous study (Dienel et al., 2020). Briefly, the mice were anesthetized with isoflurane (5% induction, 1 % maintenance). The carotid bifurcation area was then exposed and a 5‐0 nylon suture was subsequently introduced into the left external carotid artery, which was then slowly advanced along the artery, and the resistance would be felt at the anterior cerebral artery (ACA)–middle cerebral artery (MCA) bifurcation, followed by further advancement of the suture to form an endovascular perforation. Finally, the sutures were removed, and the external carotid artery was ligated. In the sham group, the sutures were advanced to the ACA–MCA bifurcation, but no endovascular perforation was made. At the 24th h after SAH, the mice were used for the detection of neurobehavioral disorders, brain edema content, blood–brain barrier (BBB) injury, inflammatory factors, and miR‐340‐5p expression.

### 2.2 Intracerebroventricular injection

The mice were randomly classified into four groups: sham group, SAH group, SAH + miR‐control group, SAH + miR‐340‐5p mimic group (18 alive mice in each group). In short, after the mice were anesthetized and fixed, 0.5 mm after bregma, a small well with a depth of 2.5–3.0 mm was opened laterally at 1 mm, and then intracerebroventricular injection was performed using a miniature syringe (Hamilton, Nevada, USA). miR‐340‐5p mimics were injected 48 h before SAH induction at a dose of 15 pmol per mouse (Sierksma et al., 2018).

### 2.3 Isolation and culture of primary neurons and microglia

The isolation of primary neurons and microglia was performed as previously described (Chen et al., 2020). Primary neurons were isolated from fetal mice. The fetal mice were decapitated, and the brain tissue was immediately obtained after peeling off the meninges and white matter. The brain tissue was then trypsinized with 0.25% trypsin (including EDTA) at 37°C for 5 min, then immersed in phosphate‐buffered saline (PBS) for three times, and the brain tissue suspension was centrifuged (1500 rpm/min, 5 min). Ultimately, the cells were resuspended in Neurobasal‐A medium with 2% B27, 2 mM L‐glutamine, 50 U/ml penicillin, and 50 U/ml streptomycin (Gibco, Carlsbad, CA, USA). Subsequent to cell counting, the neurons were inoculated in six‐well or 12‐well plates (Corning, NY, USA) covered with poly‐d‐lysine (Sigma, St. Louis, MO, USA). For primary microglia, the procedure of cell isolation is the same. Notably, the difference was the medium, which was DMEM/F12 containing 10% fetal bovine serum, 1 mM sodium pyruvate, 2 mM l‐glutamine, 100 mM nonessential amino acids, 50 U/ml penicillin, and 50 mg/ml streptomycin (Gibco). The cells were inoculated in a 150 cm^2^ flask precovered with poly‐d‐lysine (Sigma), and half of medium was refreshed every 2 days. Two weeks later, monolayer glial cells were obtained. A transwell contact‐independent neuron–microglial system was used for coculture. Neurons were inoculated in 12‐well plates (Corning, NY, USA) and microglia were seeded in transwell inserts (Corning, NY, USA). Oxyhemoglobin (OxyHb) was obtained according to a previous report (Lu et al., 2018) to stimulate the cells to establish the in vitro model of SAH. To explore the role of STING in the activation of microglia, CMA, a STING agonist (250 μg/ml), was used to treat the microglia 1 h before microglia were incubated with OxyHb (Haag et al., 2018).

### 2.4 Oligonucleotide transfection

MiR‐340‐5p mimics and their controls were provided by Thermo Fisher Scientific (Shanghai, China). At 90% cell confluence, miR‐340‐5p mimics or controls were transfected into microglia with Lipofectamine™ 2000 (Invitrogen, Waltham, MA, USA).

### 2.5 Neurological behavioral impairment

Twenty‐four hours after SAH, an independent observer, who did not know the information of the mice, used the Garcia test method to evaluate the neurobehavioral disorders of the mice (Garcia et al., 1995). Garcia test scores from 0 to 3 for each index, with a total of six indexes, including spontaneous activity, tail suspension movement of all limbs, forelimb outstretching, climbing, touching of trunk, and vibrissae touching. A total of 18 points were used to evaluate the sensorimotor dysfunction of the mice. The lower the score, the more severe the neurological impairment.

### 2.6 Evaluation of brain edema

At the 24th h after SAH, the mice were subsequently anesthetized and brain tissues were obtained, and the brainstem and cerebellum were separated, and the cerebrum was immediately weighed (wet weight, WW), and then the brain tissue was dried in an oven at 100°C for 72 h to obtain dry weight (DW). Specifically, the percentage of brain water content can be derived as (BWC) = [(WW − DW)/WW] × 100%.

### 2.7 Evaluation of BBB injury

BBB injury was evaluated as previously described (Liu et al., 2018). Briefly, 24 h after SAH, 2% Evan's blue dye (5 ml/kg; Sigma–Aldrich) was injected in the left femoral vein of the mice and circulated. One hour later, PBS was injected through the mouse heart to remove Evan's blue dye from the blood vessels of the mice. The brain samples were then collected and homogenized in 50% trichloroacetic acid, and the supernatants were collected and measured by a fluorescence spectrophotometer (excitation wavelength: 620 nm and emission wavelength: 680 nm). The findings were expressed as the amount of Evans blue per gram of brain tissue.

### 2.8 Enzyme‐linked immunosorbent assay

The concentrations of IL‐1 β, IL‐6, and TNF‐α in brain tissue and microglia medium were determined by mouse‐specific enzyme‐linked immunosorbent assay (ELISA) kits (Elabscience Biotechnology, Houston, TX).

### 2.9 Flow cytometry

The neurons were collected and the cells were rinsed with cold PBS buffer. After that, the cells were followingly resuspended in binding buffer. In each sample, 5 μl of Annexin V‐FITC staining solution and 5 μl of propidium iodide staining solution (Beyotime, Shanghai, China) were loaded, and then incubated at 4°C protected from light and stained for 30 min. The cells were subsequently rinsed three times with the binding buffer to remove excessive dye, and then resuspended in 500 μl of binding buffer. Then, the cells were detected with a BD FACSCanto II flow cytometer (BD Biosciences, San Jose, CA, USA) within 1 h, and the data were followingly analyzed by a FlowJo version 10.2 software (FlowJo, LLC Ltd, Ashland, OR, USA).

### 2.10 Quantitative reverse transcription PCR

Total RNA was extracted from brain tissue or neurons using a TRIzol kit (Invitrogen, Grand Island, NY, USA). RNA was then reversely transcribed into cDNA with SuperScriptTM III First‐Strand Synthesis System (Invitrogen, Carlsbad, CA, USA). The amplification of DNA was accomplished by a SYBR Premix Ex TaqTM II kit (TaKaRa, Dalian, China). With β‐actin and U6 as the internal references, the results were calculated by 2 ^−ΔΔCt^ formula. All primers are detailed in Table 1.

**TABLE 1:** Primer sequence used in this study

Gene	Sequence
U6	F: 5′‐ATTGGAACGATACAGAGAAGATT‐3′ R: 5′‐GGAACGCTTCACGAATTTG‐3′
Β‐Actin	F: 5′‐GCTGTCCCTGTATGCCTCTG‐3′ R: 5′‐CGCTCGTTGCCAATAGTGATG‐3′
IL‐1β	F: 5′‐TTGTTCATCTCGGAGCCTGTA‐3′ R: 5′‐AGCACCTTCTTTTCCTTCATC‐3′
IL‐6	F: 5′‐GCACTAGGTTTGCCGAGTAGA‐3′ R: 5′‐AAGCTGGAGTCAGAAGGAG‐3′
TNF‐α	F: 5′‐ATCCGCGACGTGGAACTG‐3′ R: 5′‐ACCGCCTGGAGTTCTGGAA‐3′
MiR‐340‐5p	F: 5′‐CTGGTAGGTTATAAAGCAATGA‐3′ R: 5′‐TCAACTGGTGTCGTGGAG‐3′
Caspase‐3	F: 5′‐GAAACTCTTCATCATTCAGGCC‐3′ R: 5′‐GCGAGTGAGAATGTGCATAAAT‐3′
STING	F: 5′‐ATTCCAACAGCGTCTACGAG‐3′ R: 5′‐GCAGAAGTTTAGCCTGCT‐3′

*Note*: F, forward; R, reverse; STING, stimulator of interferon genes.

### 2.11 Western blot

Cells were lysed and total protein was followingly extracted by RIPA lysis buffer (Beyotime), with the concentration evaluated by a BCA protein assay kit (Beyotime). Protein samples were followingly mixed with loading buffer, denatured, and separated by SDS‐PAGE and transferred onto PVDF membranes (Millipore, Bedford, MA, USA), which was blocked with 5% skimmed milk for 1 h at ambient temperature and incubated with the corresponding primary antibodies: anti‐Iba‐1 antibody (17198; Cell Signaling Technology, MA, USA), anti‐β‐actin antibody (8457; Cell Signaling Technology), anti‐STING antibody (13647; Cell Signaling Technology), anti‐phospho‐TANK binding kinase 1 (TBK1) antibody (5483; Cell Signaling Technology), and anti‐TBK1 antibody (3504; Cell Signaling Technology) overnight at 4°C, followed by incubation with HRP‐conjugated secondary antibody (Beyotime) for 1.5 h at ambient temperature. The protein bands were developed with an enhanced chemiluminescence kit (Beyotime). Ultimately, the relative expressions of proteins were analyzed by Image J software (NIH, USA) with β‐actin as the internal reference.

### 2.12 Lactate dehydrogenase assay

Lactate dehydrogenase (LDH) release from brain tissue as well as neurons was measured using a LDH cytotoxicity kit (Thermo Scientific, Shanghai, China) according to the manufacturer's instructions. The absorbance was measured at 450 nm wavelength with a microplate reader.

### 2.13 Luciferase reporter gene assay

The targeting relationship between miR‐340‐5p and the 3′‐UTR of STING was verified by a luciferase reporter gene assay. The wild‐type (WT) STING sequence was inserted into pmirGLO dual‐luciferase miRNA target expression vector (Promega, Madison, WI, USA) to construct the report vector pmirGLO‐STING‐WT (WT‐STING). Additionally, the 3′‐UTR of STING was mutated by a GeneArt™ Site‐Directed Mutagenesis PLUS System (cat. no. A14604; Thermo Fisher Scientific). The STING 3′‐UTR mutant (MT) sequence was inserted into the pmirGLO vector to generate the report vector pmirGLO‐STING‐MUT (MUT‐STING). Subsequently, the corresponding reporter vector and miR‐340‐5p mimic or miR‐control were cotransfected into 293T cells and then the transfected cells were cultured for 48 h. Next, the luciferase activity of the cells was detected with a Dual‐Luciferase Reporter Assay System (Promega).

### 2.14 RNA immunoprecipitation assay

The direct interaction between miR‐340‐5p and STING mRNA was verified by a Magna‐RIP‐RNA binding protein immunoprecipitation kit (Millipore Inc., Billerica, MA, USA). Briefly, microglia were lysed with RNA immunoprecipitation (RIP) buffer. The cell extract was followingly incubated with magnetic beads, which were combined with human anti‐Ago2 antibody or control IgG (Millipore Inc.). After that, the collected samples were treated with proteinase K, and then the total RNA was isolated for quantitative reverse transcription PCR (qRT‐PCR) analysis.

### 2.15 Statistical analysis

All of the experiments were performed in triplicate. All data were processed by SPSS 20.0 statistical analysis software (SPSS Inc., Chicago, IL, USA). The data were expressed as “mean ± SD” (*x* ± *s*), and one‐way ANOVA was performed for multifactor comparisons, and *t*‐test was performed for comparisons between two groups. When *p* < .05, the difference is considered to be significant.

## 3 RESULTS

### 3.1 SAH induces neurological injury, neuroinflammation, and miR‐340‐5p inhibition in mice

**FIGURE 2:**
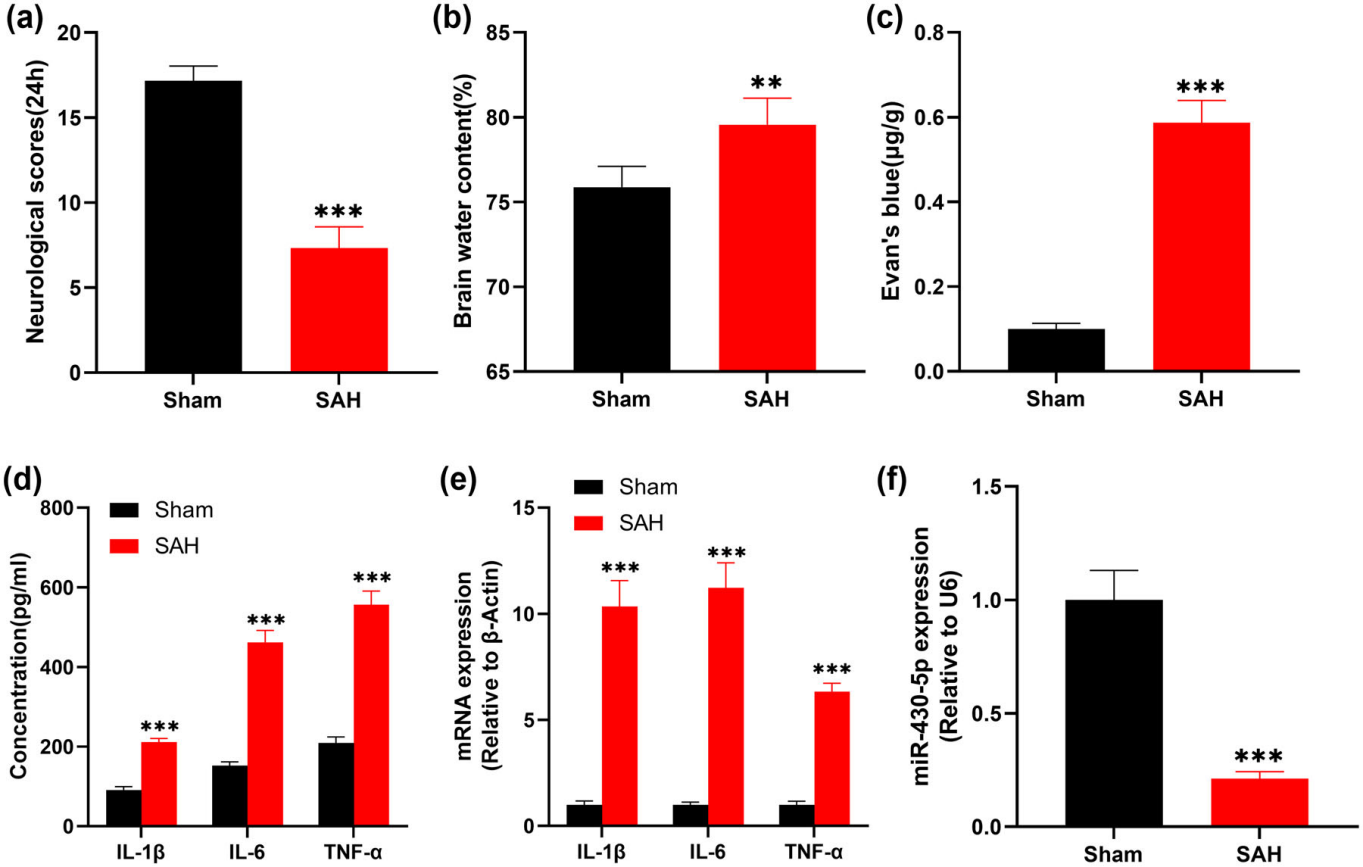
SAH causes neurological injury, neuroinflammation, and miR‐340‐5p inhibition in mice. (a) The neurobehavioral scores of mice in sham group and SAH group were evaluated by Garcia test method. (b) The brain water content of sham SAH mice was measured by dry and wet method. (c) Evan's blue staining was used to detect BBB injury of the mice. (d) The contents of IL‐1 β, IL‐6, and TNF‐α in the brain tissue of mice were determined by ELISA. (e) The expression levels of IL‐1β, IL‐6, and TNF‐α mRNA in the brain tissue of mice were assessed by qRT‐PCR. (f) The expression of miR‐340‐5p in the brain of the mice was evaluated by qRT‐PCR. *p < .01 and *p* < .001

The survival rates in sham group and SAH groups were 90% (18 out of 20) and 75% (18 out of 24). At the 24th h after SAH, there was a significant decrease in neurobehavioral scores in SAH mice compared with the sham group (Figure 2(a)), and brain edema was observed in SAH mice (Figure 2(b)), and the BBB injury was also induced in the mice with SAH (Figure 2(c)). ELISA and qRT‐PCR indicated that compared with the sham group, IL‐1β, IL‐6, and TNF‐α levels were greatly upregulated in the brain tissues of the mice in SAH group (Figures 2(d) and 2(e)). These data suggested that the model of SAH‐induced EBI was successfully established. Notably, we found lower expression of miR‐340‐5p in the brain tissues of the SAH mice as against the sham group (Figure 2F).

### 3.2 MiR‐340‐5p overexpression alleviates neural injury and neuroinflammation induced by SAH in mice

**FIGURE 3:**
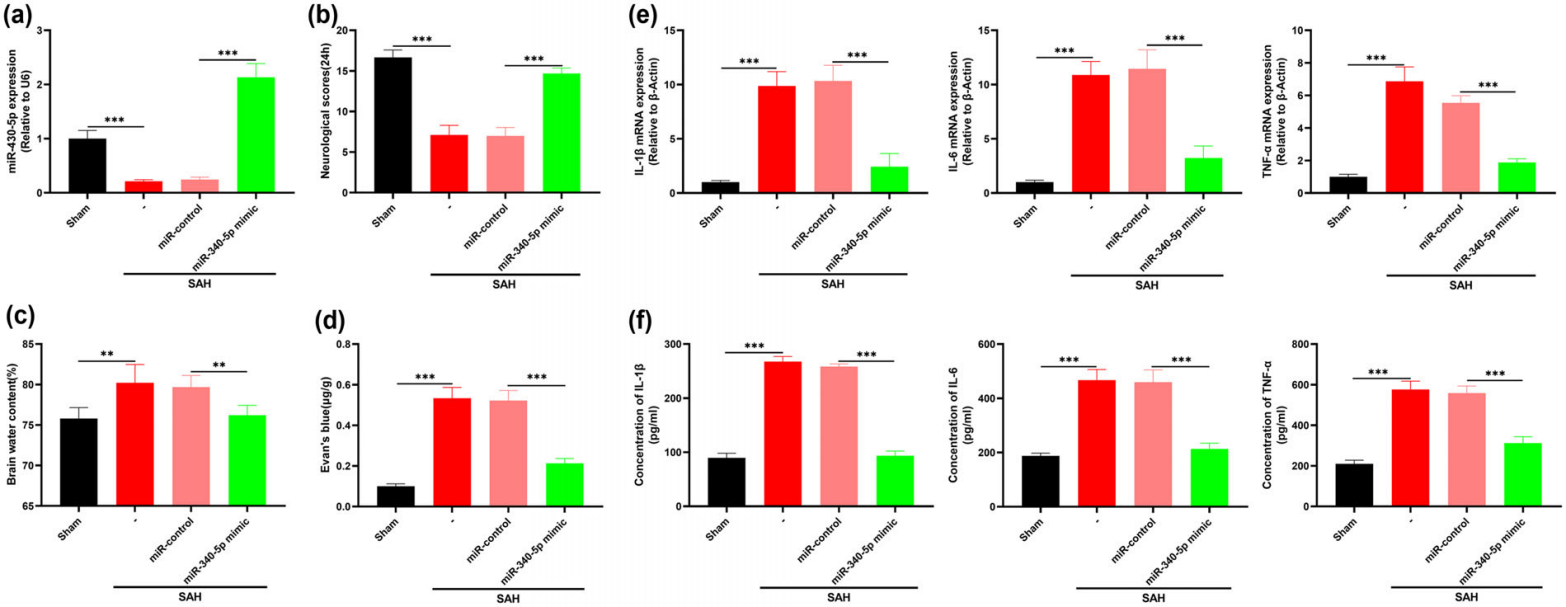
miR‐340‐5p overexpression alleviates SAH‐induced nerve injury and neuroinflammation in mice. (a) After intracerebroventricular injection of miR‐340‐5p mimic, miR‐340‐5p expressions in the sham group, SAH group, SAH + miR‐control group, and SAH + miR‐340‐5p mimic group were measured by qRT‐PCR. (b) The neurobehavioral scores of mice in sham group, SAH group, SAH + miR‐control group, and SAH+miR‐340‐5p mimic group were evaluated by Garcia test. (c) The brain water content of the mice in sham group, SAH group, SAH + miR‐control group, and SAH + miR‐340‐5p mimic group was determined by dry–wet method. (d) Evan's blue was used to detect BBB injury of the mice in sham, SAH, SAH + miR‐control, and SAH+miR‐340‐5p mimic groups. (e) The expression of IL‐1β, IL‐6, and TNF‐α in the brain of mice in sham group, SAH group, SAH + miR‐control group, and SAH+miR‐340‐5p mimic group was evaluated by qRT‐PCR. (f) The levels of IL‐1β, IL‐6, and TNF‐α in midbrain tissue of mice in sham group, SAH group, SAH + miR‐control group, and SAH+miR‐340‐5p mimic group were measured by ELISA. *p < .01 and *p* < .001

Next, we overexpressed miR‐340‐5p in mouse brain tissue by intraventricular injection of miR‐340‐5p mimics. As against the SAH + miR‐control group, miR‐340‐5p expression was markedly elevated in the brain tissue of mice with miR‐340‐5p overexpression (Figure 3(a)); the neurobehavioral score of mice was significantly increased (Figure 3(b)); the brain edema of mice was significantly reduced (Figure 3(c)); and the BBB injury of mice was significantly attenuated (Figure 3(d)). IL‐1β, IL‐6, and TNF‐α levels were significantly downregulated in mice with miR‐340‐5p overexpression as against mice in the SAH + miR‐control group (Figures 3(e) and 3(f)). Collectively, these data suggested that miR‐340‐5p overexpression could alleviate neuronal injury and neuroinflammation induced by SAH.

### 3.3 MiR‐340‐5p overexpression can alleviate OxyHb‐induced neuroinflammation and neuronal apoptosis

**FIGURE 4:**
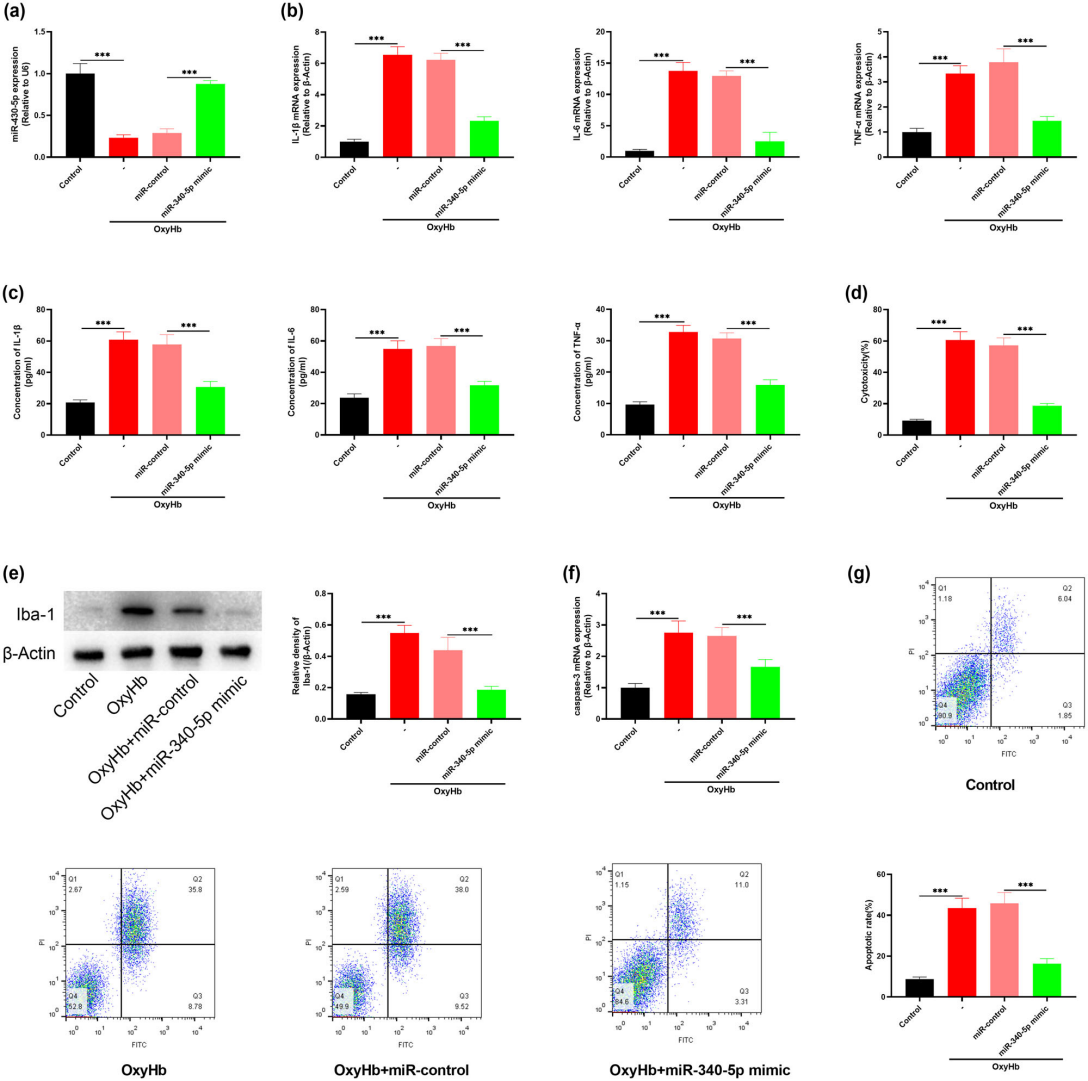
Overexpression of miR‐340‐5p alleviates OxyHb‐induced neuroinflammation and neuronal apoptosis. (a) The expression of miR‐340‐5p in microglia of control group, OxyHb group, OxyHb + miR‐control group, and OxyHb + miR‐340‐5p mimic group was determined by qRT‐PCR. (b) The contents of IL‐1β, IL‐6, and TNF‐α in microglia of control group, OxyHb group, OxyHb + miR‐control group, and OxyHb + miR‐340‐5p mimic group were detected by qRT‐PCR. (C) IL‐1β, IL‐6, and TNF‐α levels of microglia in the control, OxyHb, OxyHb + miR‐control, and OxyHb + miR‐340‐5p mimic groups were determined by ELISA. (d) LDH assay was used to assess the cytotoxicity of neurons in the control, OxyHb, OxyHb + miR‐control, and OxyHb + miR‐340‐5p mimic groups. (e) The expression of Iba‐1 in microglia of control group, OxyHb group, OxyHb + miR‐control group, and OxyHb + miR‐340‐5p mimic group was determined by Western blot. (f) The expression of caspase‐3 in neurons of control group, OxyHb group, OxyHb + miR‐control group, and OxyHb + miR‐340‐5p mimic group was determined by qRT‐PCR to assess neuronal apoptosis. (g) Flow cytometry was used to determine the rate of apoptotic neurons in the control, OxyHb, OxyHb + miR‐control, and OxyHb + miR‐340‐5p mimic groups. *p < .001

To validate the results of the experiments in vivo, we overexpressed miR‐340‐5p in microglia and exposed the microglia to OxyHb via a coculture system. Twenty‐four hours later, the effects on microglia and neurons were determined. It was observed that miR‐340‐5p was markedly downregulated in the OxyHb group compared with the normal control group; as against OxyHb + miR‐control group, miR‐340‐5p in microglia was demonstrably increased in the miR‐340‐5p overexpression group (Figure 4(a)). IL‐1β, IL‐6, TNF‐α, and Iba‐1 levels were remarkably raised in microglia of the OxyHb group compared with the normal control group; compared with OxyHb + miR‐control group, the expression of IL‐1β, IL‐6, TNF‐α, and Iba‐1 in microglia in miR‐340‐5p overexpression group was markedly suppressed (Figures 4(b) and 4(c)). As against the normal control group, LDH release in OxyHb treatment group was remarkably promoted, while compared with OxyHb + miR‐control group, the release of LDH of neurons in miR‐340‐5p overexpression group was dramatically suppressed (Figure 4(d)), suggesting that the injury of neurons was inhibited by miR‐340‐5p overexpression. Western blot suggested that the expression of Iba‐1 expression was increased in microglia after OxyHb stimulation, while reduced after miR‐340‐5p overexpression, implying that miR‐340‐5p reduced the activation of microglia. Also, the caspase‐3 expression and apoptosis of neurons in OxyHb group were significantly higher than that of the normal control group, while the effect was reversed in OxyHb + miR‐340‐5p group (Figures 4(f) and 4(g)). Collectively, it was concluded that OxyHb could activate microglia to cause neuronal inflammation and neuronal apoptosis, while miR‐340‐5p overexpression could counteract these effects.

### 3.4 MiR‐340‐5p directly regulates STING

**FIGURE 5:**
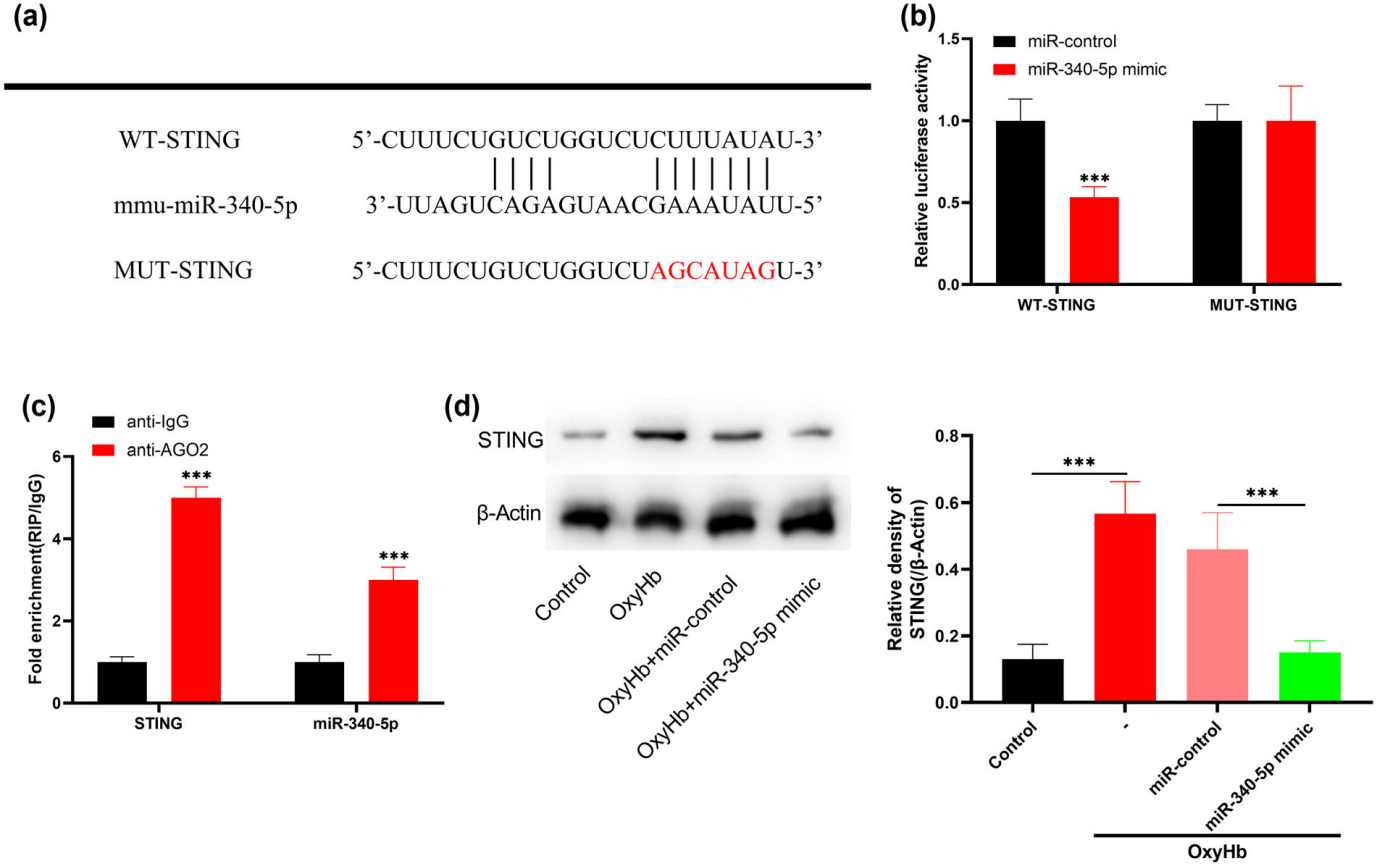
miR‐340‐5p binds directly to STING. (a) TargetScan predicted that the sequence of miR‐340‐5p matched the sequences of STING 3′ UTR. The WT‐STING and MUT‐STING luciferase reporter vectors were constructed. (b) MiR‐340‐5p mimic or miR‐control and WT‐STING or MUT‐STING were cotransfected into 293T cells. Forty‐eight hours later, luciferase activity was measured to validate the predicted binding site. (c) RIP assay was performed to verify the direct interaction between STING 3′UTR sequence and miR‐340‐5p. (d) The expression of STING in the microglia of control, OxyHb, OxyHb + miR‐control, and OxyHb + miR‐340‐5p mimic groups were detected by Western blot. *p < .001

To further explore the mechanism by which miR‐340‐5p blocks microglial activation, TargetScan 7.2 database was searched, and it predicted that there was a complementary binding site between miR‐340‐5p and the 3′UTR of STING (Figure 5(a)). Dual‐luciferase reporter gene assay showed that the transfection of miR‐340‐5p mimics greatly reduced the luciferase activity of WT‐STING reporter compared with the control mimics, but that of MUT‐STING reporter was not significantly affected by miR‐340‐5p (Figure 5(b)). RIP assay also supported that STING 3′UTR sequence could bind to miR‐340‐5p in microglia (Figure 5(c)). Western blot showed that STING expression was markedly elevated in the OxyHb group as against the control, while miR‐340‐5p overexpression significantly downregulated the expression of STING as against the OxyHb + miR‐control group (Figure 5(d)). Collectively, it was concluded that STING was a target of miR‐340‐5p in microglia.

### 3.5 Upregulation of STING can attenuate the effect of miR‐340‐5p overexpression on neurons

**FIGURE 6:**
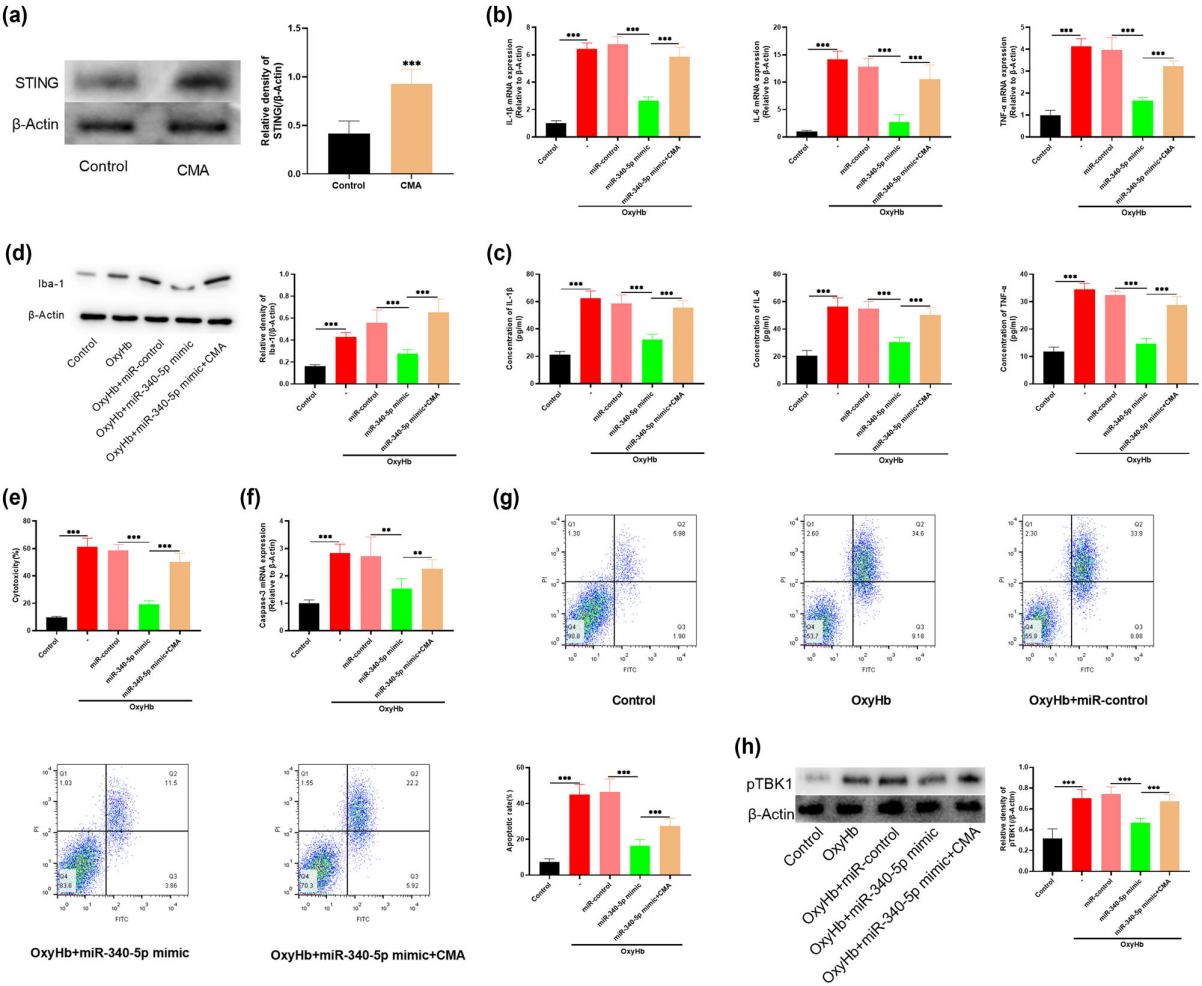
Upregulation of STING can weaken the protective effect of overexpression of miR‐340‐5p. The neurons were divided into 5 groups: Control group, OxyHb group, OxyHb + miR‐control group, OxyHb + miR‐340‐5p mimic group, and OxyHb + miR‐340‐5p mimic + CMA group. (a) Western blot was used to detect the expression of STING in microglia in control group and CMA group. (b) qRT‐PCR was used to determine the expression levels of IL‐1β, IL‐6, and TNF‐α in microglia of each group. (c) The levels of IL‐1β, IL‐6, and TNF‐α in the supernatant of microglia in each group were determined by ELISA. (d) The expression of Iba‐1 in microglia of each group was detected by Western blot. (e) LDH assay was used to determine the injury of neurons in each group. (f) The expression of caspase‐3 in neurons of each group was determined by qRT‐PCR. (g) The level of neuronal apoptosis in each group was measured by flow cytometry. H. Phosphorylation levels of TBK1 in microglia of each group were determined by Western blot. *p < .01, and *p* < .001

To further validate the role of STING in the microglial inflammatory responses, we treated microglia with 250 μg/ml of the STING agonist CMA. It showed that STING expression was greatly raised in microglia after CMA treatment compared with the control group (Figure 6(a)). After STING activation, the levels of IL‐1β, IL‐6, TNF‐α, and Iba‐1 in microglia were significantly upregulated; neuronal cytotoxicity, caspase‐3 expression, and apoptosis of neurons were significantly enhanced, compared with the neurons cultured with microglia with miR‐340‐5p overexpression (Figures 6(b)–6(g)). Additionally, it was revealed that the phosphorylation of TBK1, a downstream protein of STING signaling, was also promoted after OxyHb treatment, but repressed after miR‐340‐5p overexpression (Figure 6(h)). Collectively, it was concluded that miR‐340‐5p attenuated OxyHb‐induced neuroinflammation and neuronal injury via inhibiting the STING signaling pathway.

## 4 DISCUSSION

Abnormal activation of microglia mediates neuroinflammation and partakes in the pathogenesis of neurological diseases, such as Alzheimer's disease, ischemic stroke, brain trauma, and so on (Gris et al., 2019). Neuroinflammation not only aggravates EBI, but also plays an important pathological role in the secondary injury of SAH (Khey et al., 2020; Schneider et al., 2018). As reported, inhibition of neuroinflammation can significantly ameliorate neurological injury after SAH. For example, fluoxetine inhibits neuroinflammation through regulating TLR4/MyD88/NF‐κB signal pathway in a rat model with SAH and improves the neurological functions of the rats (Liu et al., 2018); Aggf1 inhibits neuroinflammation in a rat model with SAH through PI3K/Akt/NF‐κB pathway to repress EBI (Zhu et al., 2018). Notably, miRNAs act as important regulators in multiple biological processes and many of them are involved in regulating neuroinflammation (Al‐Ghezi et al., 2019). It is reported that, miR‐210 promotes neuroinflammation in neonatal hypoxic‐ischemic encephalopathy (Li et al., 2020); miR‐146a prevents cognitive decline induced by surgical trauma by inhibiting neuroinflammation (Chen et al., 2019). It has been reported that miR‐340‐5p mitigates oxygen‐glucose deprivation/reoxygenation‐induced neuronal injury through targeting neuronal differentiation 4 (Zheng et al., 2020). Additionally, miR‐340‐5p targets PDCD4 to reduce inflammatory response and ameliorate intracerebral hemorrhage‐induced brain injury (Zhou et al., 2020). Also, miR‐340‐5p mitigates spinal cord injury‐induced neuroinflammation and neuronal apoptosis via modulating the p38‐MAPK signaling pathway (Qian et al., 2020). These studies suggest that miR‐340‐5p is a crucial regulator in neuroinflammation and plays a protective role. Consistently, the present study confirms that, in vivo and in vitro, miR‐340‐5p expression was reduced after SAH, and miR‐340‐5p restoration/overexpression significantly inhibited microglia activation and neuroinflammation and protect neurons.

This study also reports that STING is a downstream target of miR‐340‐5p. STING serves as a vital intracellular signaling molecule, sensing pathogens, and regulating innate immunity. Previous studies have shown that activated STING recruits and phosphorylates TBK1, which in turn activates IFN regulatory factor 3, ultimately elevating type I IFN expression and promoting the inflammatory response by recruiting and activating immune cells, and STING is considered as a protector against pathogens (Hu & Shu, 2020). Notably, however, abnormal activation of STING signaling leads to tissue injury. For instance, excessive activation of STING in liver is associated with the progression of nonalcoholic fatty liver disease by strengthening macrophage‐mediated liver inflammation and fibrosis (Qiao et al., 2018); STING pathway regulates the activation of NLRP3 inflammasome in bone marrow cells and cardiomyocytes (Gaidt et al., 2017). It is also reported that activation of STING is closely associated with neuroinflammation (Paul et al., 2021). Here, we report that miR‐340‐5p directly inhibits STING expression in microglia, decreases the phosphorylation level of TBK1, and suppresses neuroinflammation. Our data partly explain the mechanism by which miR‐340‐5p regulates the neuronal injury in SAH.

Our data suggest that restoration of miR‐340‐5p is a promising strategy to treat some inflammation‐related neurological diseases including SAH. However, intraventricular injection of miR‐340‐5p is invasive, and it is not suitable for clinical application. It is necessary to look for a more practical and safe way to selectively regulate miR‐340‐5p in vivo. In the present work, we focused on the part of miR‐340‐5p/STING axis in modulating microglia‐mediated neuroinflammation after SAH. Instead, whether miR‐340‐5p modulates other targets/pathways to exert protective effects, is still unclear, which require follow‐up investigation.

In summary, in SAH, miR‐340‐5p exerts a neuroprotective role by inhibiting STING expression, thereby inhibiting microglia activation, attenuating neuroinflammation, and reducing neuronal apoptosis. Our study provides a novel mechanism of the pathogenesis of EBI in SAH and offers clues for the treatment of SAH.

## CONFLICT OF INTEREST

The authors declare that they have no competing interests.

## FUNDING

This research received no specific grant from any funding agency in the public, commercial

### PEER REVIEW

The peer review history for this article is available at https://publons.com/publon/10.1002/brb3.2687

## Data Availability

The data used to support the findings of this study are available from the corresponding author upon request.
